# Characterizing Task-Based OpenMP Programs

**DOI:** 10.1371/journal.pone.0123545

**Published:** 2015-04-10

**Authors:** Ananya Muddukrishna, Peter A. Jonsson, Mats Brorsson

**Affiliations:** 1 KTH Royal Institute of Technology, Stockholm, Sweden; 2 SICS Swedish ICT, Stockholm, Sweden; Mathematical Institute, HUNGARY

## Abstract

Programmers struggle to understand performance of task-based OpenMP programs since profiling tools only report thread-based performance. Performance tuning also requires task-based performance in order to balance per-task memory hierarchy utilization against exposed task parallelism. We provide a cost-effective method to extract detailed task-based performance information from OpenMP programs. We demonstrate the utility of our method by quickly diagnosing performance problems and characterizing exposed task parallelism and per-task instruction profiles of benchmarks in the widely-used Barcelona OpenMP Tasks Suite. Programmers can tune performance faster and understand performance tradeoffs more effectively than existing tools by using our method to characterize task-based performance.

## Introduction

OpenMP is a popular parallel programming API where programmers express logical units of parallelism which are scheduled on threads by a runtime system. Standardization, wide-spread compiler support and quick, incremental parallelization features are among the reasons behind the popularity of OpenMP. OpenMP 3.0 introduced tasks as an explicit notion of logical parallelism to simplify expressing irregular and nested parallelism in a composable manner [1].

However, the relative ease of expressing irregular and nested parallelism using tasks does not simplify solving performance problems in OpenMP. Exposing enough task parallelism to maximize memory hierarchy utilization while simultaneously minimizing parallelization overheads is crucial for performance. Memory hierarchy utilization and parallelization overheads can be inferred using thread state performance provided by current state-of-the-art OpenMP tools [2–7]. Understanding task-based performance such as exposed task parallelism and execution of per-task instances is a struggle since tools provide limited support for tasks, report primarily thread-centric performance and do not close the semantic gap between tasks and threads [4]. Only expert programmers are able to cope by probing the application to reveal tasks and wading through the parallelization mechanics of the compiler and the runtime system in order to manually infer task-based performance.

Moreover, improving program performance by balancing task parallelism with memory hierarchy utilization is an iterative process. Code and scheduling modifications alter task-based composition. The newly exposed task parallelism has to be re-understood tediously for each iteration. The number of iterations spent in the performance tuning process varies depending on programmer experience, but multiple iterations are typically necessary even for experienced programmers.

Even simple task-based programs are surprisingly difficult to debug. Consider the task-based Fibonacci program which spans a few lines of code and whose sequential execution is well understood. Parallel performance remains poor despite solving thread-based performance problems such as load imbalance and contention pointed out by tools. The root of the problem is ill-suited task granularity which tools do not point out. Programmers resort to expert help or understand task granularity by tedious code and system inspection. Lack of task-based performance information compounds debugging difficulty for complex programs and those without source code.

Tool support for directly understanding task-based performance eliminates the step of manual inference in debugging performance problems. The iterative balancing process can be short-circuited allowing programmers to test changes to their programs and immediately understand the effects of the changes. Better tools help programmers regardless of their experience—inexperienced programmers can begin to approach and understand performance issues and experienced programmers will identify and resolve performance issues faster.

Designing the necessary tool support is challenging since there is an inherent conflict between performance of the tools and the quality of the information collected. We contribute with a tool design that provides rich task-based performance information at manageable costs. To obtain detailed task-performance, our tool combines binary instrumentation and hardware performance counter readings captured during task events. We demonstrate utility by using our tool to characterize architecture independent task-based performance of benchmarks in the Barcelona OpenMP Tasks Suite (BOTS) [8] in extensive detail. Our characterization provides input sensitivity and similarity of BOTS benchmarks for the first time to aid benchmark development and task scheduling research in the OpenMP community. Furthermore, we demonstrate how task-based metrics can be used to diagnose performance problems in OpenMP programs quickly and more effectively than what is possible using thread-based metrics provided by existing tools.

Our contributions are:

- We describe a simple automated method to extract task-based performance in OpenMP programs.
- We apply the method to produce an extensive, architecture independent characterization of task-based performance of BOTS [8].
- We demonstrate how task-based performance can be used to diagnose performance problems quickly and understand performance tradeoffs in OpenMP programs.

## Need For Task-based Performance Analysis

We demonstrate the usefulness of task-based performance analysis by providing a more detailed explanation of the Fibonacci problem mentioned in the Introduction section. The Fibonacci program we consider is part of BOTS. Despite its simplicity, BOTS Fibonacci requires an input called *depth cutoff* for performance. The depth cutoff controls the granularity of tasks and is commonly provided as the -x argument during program invocation. Since thread-based performance measurements such as speedup and thread state durations do not show per-task granularities, programmers are forced to find the best performing depth cutoff by manual tuning.

**Fig 1:**
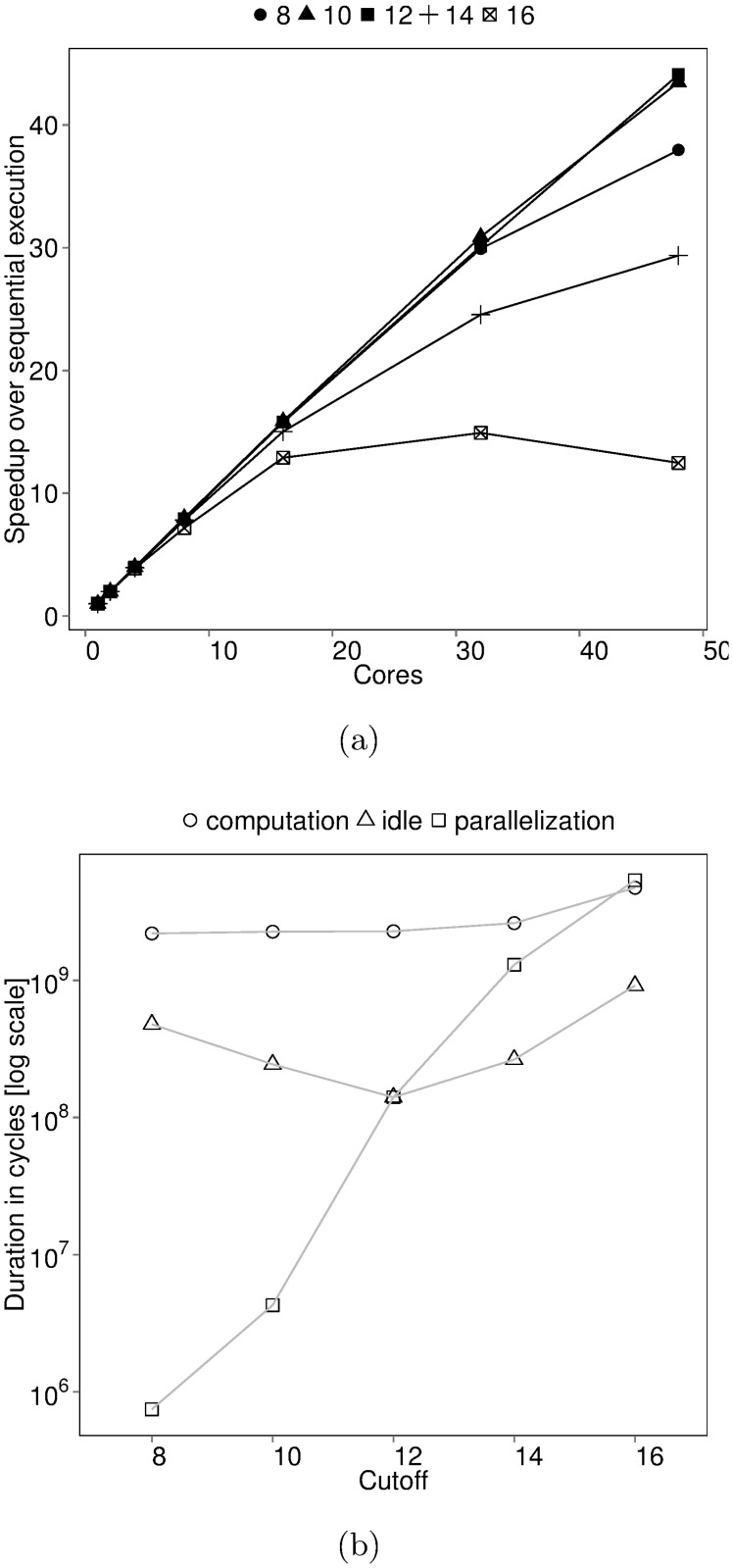
Thread-based performance of BOTS Fibonacci. Input: n = 48, depth cutoff = {8,10,12,14,16}. Executed on 48 cores of a machine with four 12-core AMD Opteron 6172 processors running at highest frequency with frequency scaling turned off. The task scheduler used balances load using thread-private task queues and random work-stealing. (a) Speedup (b) Average number of processor cycles spent in thread states. Threads create and synchronize tasks in the *parallelization* state, execute tasks in the *computation* state and enter the *idle* state when work cannot be found.

Consider a manual tuning session where BOTS Fibonacci is executed with inputs *n* = 48 and depth cutoffs = {8, 10, 12, 14, 16} on a 48-core machine with four AMD Opteron 6172 processors. Speedup measured during the tuning session is shown in Fig 1a. We refer to the depth cutoff simply as cutoff through the remainder of the section. A cutoff of 12 gives the best performance. Choosing a proper cutoff is crucial for Fibonacci performance.

Let us investigate the sensitivity of Fibonacci performance to the cutoff value. Speedup measurements indicate the impact of cutoff on performance but cannot explain why. Thread state durations cannot adequately explain the cutoff performance either. We show trends in the average time spent in different threads states in Fig 1b. Performance is worst at cutoff 16 since threads spend more time in parallelization than in execution. However, lower cutoffs also perform poorly despite good parallelization to execution time ratios. In addition, we cannot explain why overall parallelization time is high. We use idle state time as a proxy indicator of load-balance to conclude that cutoff 12 provides the highest core utilization. However, we cannot determine the reason behind the uneven load. Also, the slight increase in execution time with cutoff is puzzling since work is constant (input n = 48) during tuning.

Speedup and thread state durations are thread-based metrics that are useful when programmers use threads to compose programs. However, while composing task-based programs, programmers think about tasks without concern for threads and scheduling. Thread-based metrics explain task-based program performance poorly since they fail to probe execution at the level of tasks understood by programmers. Yet thread-based metrics are the only support available from state-of-the-art performance analysis tools.

Lack of in-depth performance diagnosis support from existing tools encourages a culture of ignorance among programmers. Only expert programmers are able to probe deeper than tools and explain performance at the cost of manual probing time. When expert understanding is absent, manual tuning is the sole resort. Manual tuning suffers from combinatorial explosion for task-based programs that have more than one tuning parameter. A majority of BOTS benchmarks have multiple (up to four) cutoffs which expand tuning space to a huge and unmanageable extent.

**Fig 2:**
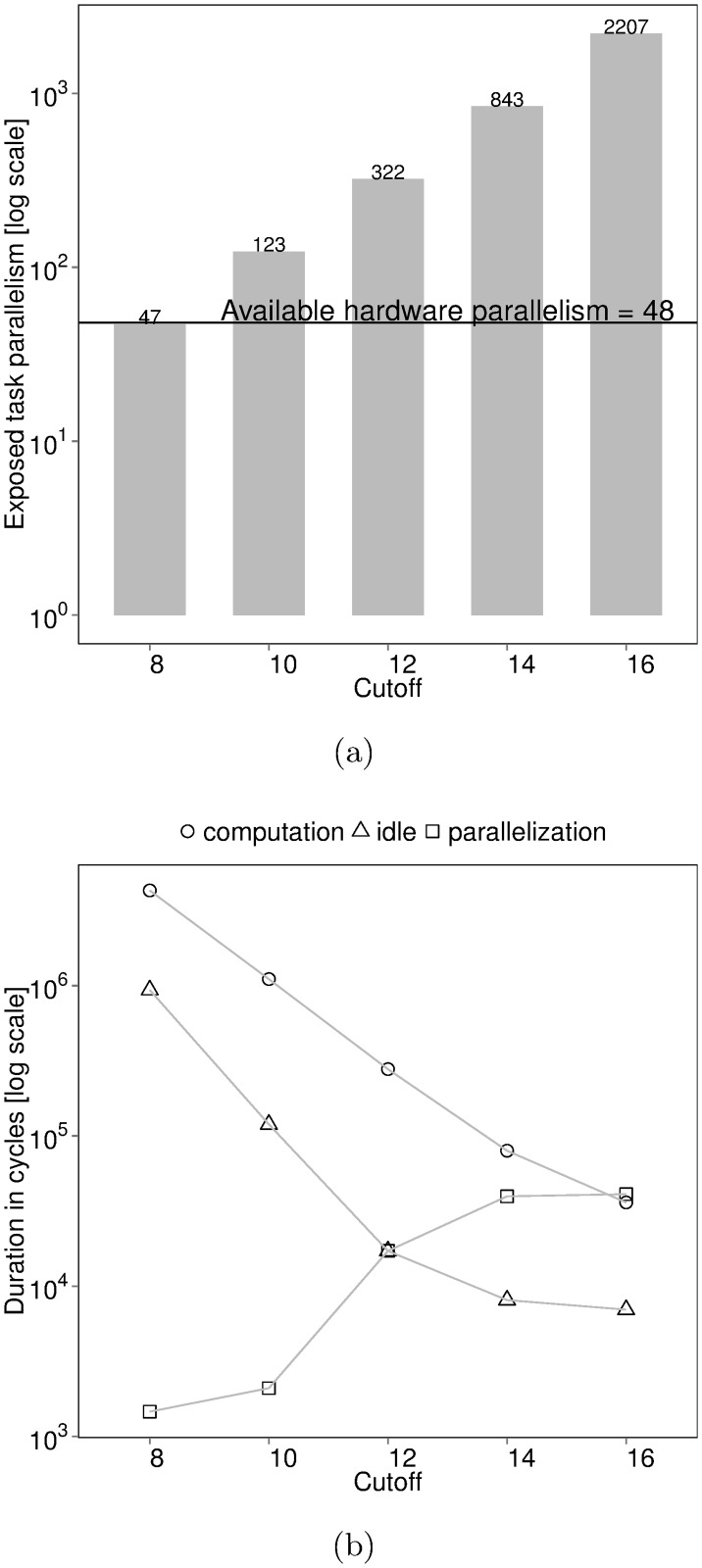
Task-based performance of BOTS Fibonacci. Input: n = 48, depth cutoff = {8,10,12,14,16}. Executed on 48 cores of a machine with four 12-core AMD Opteron 6172 processors running at highest frequency with frequency scaling turned off. The task scheduler used balances load using thread-private task queues and random work-stealing. (a) Task parallelism exposed during execution. We calculate task parallelism by dividing total task computation by the length of the critical path of the task graph. The horizontal line indicates the available 48-core hardware parallelism. (b) Average processor cycles *per task* spent in thread states. Threads create and synchronize tasks in the *parallelization* state, execute tasks in the *computation* state and enter the *idle* state when work cannot be found.

Task-based metrics can explain Fibonacci performance conclusively. Fig 2a shows exposed task parallelism for different cutoffs. Exposed task parallelism is a first-class task-based performance metric obtained from the shape of the task graph. We divide the total number of execution cycles of all tasks by the number of execution cycles on the critical path of the task graph to infer exposed task parallelism, as per the method established by Cilk theory [9]. A cutoff value of 8 performs poorly since exposed task parallelism is less than the available hardware parallelism. We can conclude that higher cutoffs lead to more parallelism.

*Per-task* time, yet another first-class task-based performance metric, sheds further light on Fibonacci cutoff performance. Per-task time is obtained by measuring thread state time at the granularity of tasks and is shown in Fig 2b. Increasing cutoff decreases both per-task execution and idle state time. Higher cutoffs lead to more fine-grained tasks in turn increasing parallelization costs. A cutoff of 16 creates many fine-grained tasks which take longer to parallelize than execute. Large-grained tasks limit parallelism at lower cutoffs despite low parallelization costs. The increasing execution state time in Fig 1b can now be blamed on a stressed runtime system and poor memory hierarchy utilization—a phenomenon called *work time inflation* [10].

Per-task time in Fig 2b guides the choice of good cutoffs for Fibonacci. The performance *sweet-spot* is the intersection between the per-task idle trend line and the parallelization trend line. Parallelization cost is balanced with load-balancing at the sweet-spot. The intersection between the per-task parallelization trend line and the execution trend line indicates where the runtime system has turned into a bottleneck.

Using task-based performance metrics, we have successfully reverse-engineered the influence of cutoff on Fibonacci performance. Such insight was impossible using thread-based metrics alone. Thread-based metrics can show how well a program performs under different core counts and point out load balance problems so they are excellent indicators of scalability. However, they fail to show problems in exposed task parallelism and per-task execution solving which is crucial for task-based program performance. Therefore tools should provide task-based metrics in addition to those based on threads for meaningful and quick performance analysis of task-based programs.

## Characterizing Task-based Performance

We describe an automated method to extract detailed task-based performance information from OpenMP programs at manageable costs in this section. We first classify task-based performance and provide background information on source translation and runtime system execution of OpenMP tasks. Next, we explain the details of our performance extraction method.

### Terminology

We consider independent (not derivable from one another) task-based performance metrics and classify them into one of:

- **Task graph properties** describe the task-based composition of the program and are shown in Table 1. Task graph properties are typically architecture independent but can become architecture dependent in non-deterministic programs.
- **Per-task properties** describe individual tasks of the program and are shown in Table 2. Per-task properties allow the study of tasks in isolation.

**Table 1:** Task graph properties.

**Property**	**Description**
Number of tasks	Number of tasks created by the program.
Inlined execution	Tasks inlined dynamically by the runtime system [11].
Fork-join structure	The task graph indicating parent and child task relationships.
Critical path	The length and tasks part of the longest path in the task graph.
Exposed task parallelism	Total execution time of all tasks divided by total execution time of tasks on the critical path. The metric is also a loose upper bound estimate on the number of parallel resources needed for program execution [9].

**Table 2:** Architecture independent per-task properties.

**Property**	**Description**
Instruction count	Number of dynamic instructions executed.
Instruction mix	Categorical number of dynamic instructions executed.
Stack reads	Number of read accesses to stack memory.
Stack writes	Number of write accesses to stack memory.
Memory reads	Number of read accesses to main memory (excl. stack).
Memory writes	Number of write accesses to main memory (excl. stack).
Memory footprint	Number of addresses accessed from main memory (excl. stack).
Computational intensity	Instruction count per memory operation (both read and write, excl. stack).
Source location	Location in original and translated source code.
Data environment	Data values inherited.

We focus on architecture independent task properties since our goal is to characterize task-based performance inherent in the program. However, our method also provides architecture dependent task properties such as per-task execution time and memory hierarchy statistics which are useful for performance analysis.

Note that we refer to code dependent, micro- and memory-architecture independent properties as architecture independent per-task properties in the paper. We classify instruction count and instruction mix as architecture independent per-task properties in Table 2 in a general sense although they are not independent of ISA.

Tasks in OpenMP are defined by surrounding code regions using the *task* statement. OpenMP source translators typically handle task definitions by moving the surrounded code region into a named function and inserting an asynchronous call in its place. The named function is called the *outline function*. The asynchronous function call is made using the *task creation* interface provided by the runtime system.

The runtime system assigns an unique identifier to each newly created task. The creating task is called the *parent* and the created task the *child*. The point at which the child task is created is called a *fork* point in the execution of the parent task.

OpenMP tasks synchronize using the *taskwait* statement. The parent task waits for all child tasks to finish execution during task synchronization. The point at which child tasks are synchronized is called a *join* point in the execution of the parent task. Source translators handle synchronization using the *task synchronization* interface provided by the runtime system.

### Extraction Method

**Fig 3:**
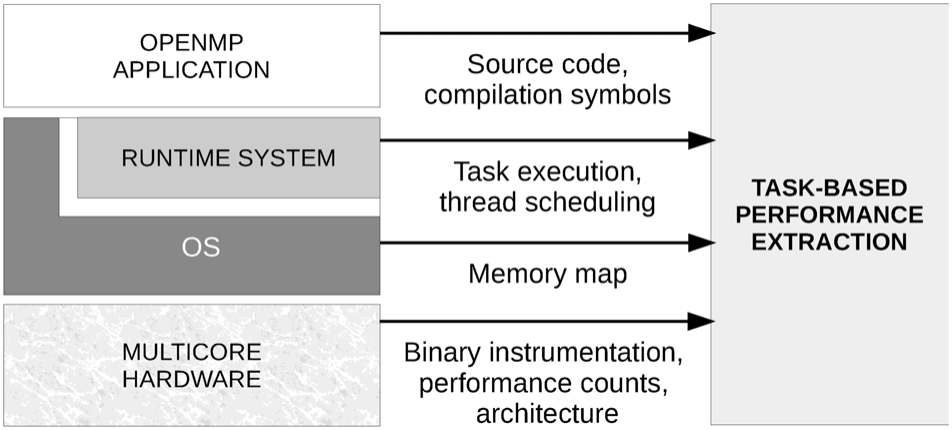
Extraction of task-based performance from OpenMP programs.

The extraction method combines information from source translation, native compilation, runtime system task management, OS memory management, dynamic binary instrumentation, hardware performance counters and architectural topology to derive task-based performance information as shown in Fig 3.

We extract task graph properties using task creation and synchronization information from the runtime system. Task identifiers, data environment and scheduling point timestamps are recorded during extraction.

We extract per-task properties by uniquely identifying task instances and recording instructions and hardware performance counters during task execution.

#### Tracking task instances

We use a contextual stack-based approach to track task execution. A new context is created when a task begins execution. The previous context is pushed on the stack and the newly created context is set as current. All profiling costs are attributed to the current context. The context on the stack is restored when the current task finishes execution.

Our approach to track task execution is simple to implement and requires two things. Inlining calls to the task outline function during compilation must be disabled to correctly distinguish task contexts. Disabling inlining preserves the profiled task properties. Second, costs of dynamically inlined tasks [11] must be transferred to the parent context in a post-profiling step since inlined tasks appear as real tasks to the profiler.

#### Obtaining per-task properties

We obtain per-task instructions using dynamic binary instrumentation. We obtain per-task execution and memory hierarchy performance by capturing hardware performance counter values at task execution boundaries. Code executed within outline functions including nested user-level function calls are included while counting performance. Runtime system function calls made from the task context are excluded to avoid misattribution of parallelization activity to task execution. We link executing tasks to source code using compilation information (Example: AST using GCC option *-fdump-tree-optimized*).

Note that we minimize performance skewing by collect binary instrumentation and hardware performance counter information during mutually-exclusive profiling steps. Binary instrumentation runs once since architecture independent performance remains constant for a given program-input pair. Hardware performance collection repeats several times to filter noise and minimize multiplexing costs incurred while reading multiple counters simultaneously.

#### Post-processing

We process the extracted task graph properties to construct the fork-join task graph and associate per-task properties with graph elements. We also plot the task graph to visualize how program tasks were created at runtime.

Tasks on the critical path of the task graph are good optimization candidates [12]. We obtain the critical path of the task graph using individual task instruction count as a proxy for architecture independent execution time.

We additionally derive the amount of exposed task parallelism using the critical path and total instruction count of all tasks. Our derivation is based on the notion of *logical* parallelism in the Cilkview tool [9]. Exposed task parallelism can quickly explain under-utilization of available hardware parallelism (cores) as demonstrated in the Fibonacci example in the previous section. Exposed task parallelism also provides a loose upper bound on the amount of hardware parallelism required for program execution [9].

We estimate the computational intensity of tasks by measuring the number of instructions executed per memory read or write operation. Computational intensity is a useful metric for guiding the choice of performance optimizations—a concept elegantly demonstrated by the Roofline model [13].

### Prototype Implementation

We built a prototype tool that implements the extraction method using Intel’s Pin-2.12 [14] for binary instrumentation, PAPI [15] for accessing hardware performance counters, R [16] for data-parallel post-processing and igraph [17] for task graph plotting.

We tested the tool on task-based OpenMP programs compiled using GCC-4.8.0 with optimization O2, linked with a custom runtime system called MIR [18] and executed on Linux-kernel-2.6.32. Implementing tool functionality in other runtime systems require two simple extensions—a mechanism for unique identification of task instances and a mechanism for attributing hardware and instruction-level performance to each task.

We disabled inlining of functions in program code to enable our method to distinguish task contexts as explained before. Disabling inlining degraded performance by less then 8% for BOTS benchmarks.

We cannot distinguish and exclude runtime system data structure operations while instrumenting tasks due to profiling infrastructure limitations. We compensate by subtracting runtime system data structure operations—typically constant—from profiled information in the post-processing step. We additionally cannot instrument dynamically linked user-level function calls and system calls made in the context of outlined functions due to profiling infrastructure and OS protection limitations. We account for system calls in the post-processing step and statically link user-level functions whenever possible.

Our tool minimizes required compiler and runtime system support by ignoring implicit tasks, untied tasks and tasks which live beyond their parent. Implicit tasks can be tracked by defining tasks explicitly in the *parallel* region. Untied tasks can be tracked with additional implementation complexity and is being considered for future versions of the tool. Tasks which live beyond the parent, also called *rogue tasks*, are uncommon, problematic to profile, and will likely be removed in future OpenMP specifications to improve composability. Our tool also minimizes instrumentation overheads by disregarding program performance outside the scope of tasks.

We have built our tool such that performance depends on desired profiling information richness. The task graph structure is profiled in parallel with less than 1% overhead. Reading cycle and cache stall counters to obtain architecture dependent per-task properties, also done in parallel, incurs an average 2.5% overhead for BOTS benchmarks. Profiling architecture independent per-task properties—task instructions, memory footprint and memory accesses—slows execution by 36X for BOTS benchmarks. The slowdown is mainly due to sequential profiling and unavoidable binary instrumentation technology overheads. We use sequential profiling to simplify our Pin tool implementation which uses a single task-tracking stack and avoids complicated book-keeping or locking. However, the relatively high cost to obtain architecture independent per-task properties is paid only once since the properties remain constant for a given program-input pair. Once profiled, architecture independent per-task properties can be carried across runs with different core allotments (available hardware parallelism). Furthermore, low overhead profiling of the task graph and architecture dependent per-task properties permits analysis of long-running programs.

## BOTS Characterization

The Barcelona OpenMP Tasks Suite [8] (BOTS) is a set of task-based OpenMP benchmarks used to evaluate task-based OpenMP implementations. BOTS is composed of the following benchmarks: Alignment, FFT, Fibonacci, Floorplan, Health, NQueens, Sort, SparseLU and UTS. Alignment and SparseLU are iterative while the rest are recursive.

We contribute with a detailed architecture independent task-based characterization of BOTS benchmarks to complement existing predominantly thread-based characterizations. We first describe the relationship between benchmark inputs and exposed task parallelism. Next, we analyze benchmark input sensitivity to architecture independent task performance. We describe the similarity between benchmarks by comparing task graph and per-task properties across inputs in the end.

### Inputs

**Fig 4:**
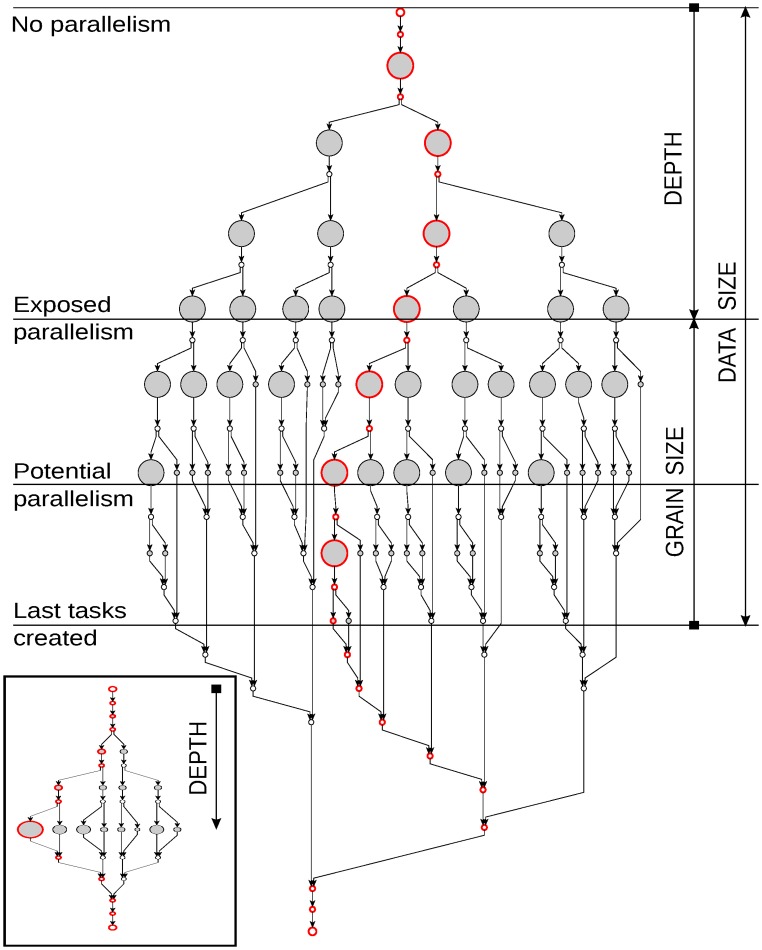
Task graph of Fibonacci. Large graph: input n = 8, depth cutoff = ∞. Small graph in inset: input n = 8, depth cutoff = 3. Size of task nodes (gray) indicates instruction count. Critical path is marked by nodes with a red border.

Understanding the influence of inputs is necessary to properly evaluate BOTS benchmarks because the inputs control both data size and task parallelism. We characterize the influence of inputs on exposed task parallelism by first classifying the inputs into DATA SIZE, GRAIN SIZE and DEPTH. Fig 4 shows the relationship between the inputs using the iceberg-shaped task graph (plotted by our tool) of BOTS Fibonacci—a typical example of recursive fork-join programs.

The task graph shows tasks using gray colored circles. The size of the task circles encodes instruction counts—large-grained tasks appear as large circles. Fork-join points are indicated using plain circles. The longest path of the graph—critical path —in terms of instruction count is marked by circles with a red border.

The task graph begins execution at the top with a single root task which divides the Fibonacci problem and branches off to solve the parts in parallel. Each branch divides the problem further exposing more parallelism and widens the task graph. The extent of division in a branch is proportional to the problem specified by the DATA SIZE input.

The task graph becomes widest when most branches cannot divide the problem further. The highest amount of task parallelism—the *potential parallelism* of the program—is exposed at the widest part. Performance is limited when potential parallelism is reached since instruction count of tasks—grain size—is small and comparable with parallelization overheads. The problem is solved by limiting branch growth to a depth where task grain sizes are practical for performance. The task graph with limited branch growth now contains the *exposed parallelism* of the program at the widest point. The growth-limited task graph of Fibonacci is shown in the inset in Fig 4.

Both GRAIN SIZE and DEPTH inputs shown in Table 3 are used to limit branch growth. GRAIN SIZE specifies the size of the problem at which branches should stop division. DEPTH specifies the maximum depth to which a branch can grow. GRAIN SIZE and DEPTH are commonly called *cutoff* parameters in BOTS. Tasks at the cutoff point (end of branch) are called *leaf* tasks which typically execute the brunt of the work.

**Table 3:** Inputs to limit task graph size in BOTS benchmarks.

**Input**	**Benchmarks**
GRAIN SIZE	Alignment, FFT, Sort, SparseLU, Strassen
DEPTH	Fibonacci, Floorplan, Health, NQueens, Strassen, UTS

Potential and exposed parallelism can also be seen in iterative fork-join programs such as Alignment and SparseLU in BOTS. The task graph of iterative fork-join programs contains a distinct spine which represents the iterations made to solve the problem. The length of the spine is proportional to the problem specified by the DATA SIZE input. Tasks created within an iteration spread out horizontally from the spine with grain sizes proportional to the width of the spread. The widest spread of tasks indicates the potential parallelism of the program. Tasks on the widest spread have instruction counts lower than parallelization overheads which limits performance. The problem is solved by curbing the spread which reduces potential parallelism to exposed parallelism. The GRAIN SIZE input curbs spreading by specifying the finest possible task grain size. The DEPTH input is not applicable since the notion of depth does not exist in the task graph of iterative fork-join programs.

### Input Sensitivity

We measure BOTS input sensitivity by quantifying changes in task graph and architecture independent per-task properties with increasing data sizes. We use input sensitivity results to validate input behavior explained in the previous section and estimate scalability of benchmarks.

We found it difficult to choose standard and meaningful input values while designing our input sensitivity experiment. BOTS contains inconsistent and outdated input values recognizing which widely varying input values have been used in existing studies. In addition, cutoff values chosen for experiments are rarely indicated. We solved the problem by choosing input values to match the most common experiment machine size.

Our experimental setup to measure input sensitivity was as follows. We chose three increasing data sizes and a constant cutoff such that each benchmark executed for 1, 5 and 10 seconds on all cores of a 24-core AMD Opteron 6172 machine with frequency scaling turned off. We chose 24 cores to reflect the most accessible machine size for researchers. The three data sizes and constant cutoffs chosen for each benchmark are respectively shown in Tables 4 and 5. We profiled each benchmark using our prototype tool and extracted task graph and per-task properties for the three data sizes.

**Table 4:** Data sizes to study BOTS input sensitivity.

**Benchmark**	**Input**	**1s**	**5s**	**10s**
Alignment	Number of sequences	100	200	400
FFT	Number of samples	2^21^	2^23^	2^24^
Fibonacci	Number	48	50	52
Floorplan	Number of shapes	10	15	20
Health	Cities	22	28	34
NQueens	Number of queens	13	14	15
Sort	Array size	2^24^	2^26^	2^27^
SparseLU	Number of blocks	64	96	128
Strassen	Matrix size	2048	4096	8192
UTS*	Root branching factor	10000	30000	50000

* UTS is a synthetic stress benchmark whose default inputs produce an extraordinary amount of tasks—approx. 1.5–4 billion—which cannot be profiled using our system. We have chosen input sets for UTS which produce approx. 100–300 thousand tasks and maintain stress.

**Table 5:** Inputs held constant while studying BOTS input sensitivity.

**Benchmark**	**Input**	**Value**
Alignment	None	NA
FFT	None	NA
Fibonacci	Depth cutoff	14
Floorplan	Depth cutoff	7
Health	Levels, Population ratio, Time, Assess time, Convalescence time, Seed, Get sick probability, Convalescence probability, Reallocation probability, Depth cutoff	4, 10, 30, 2, 12, 23, 0.002, 0.1, 0.15, 2
NQueens	Depth cutoff	3
Sort	Quicksort cutoff, Insertion sort cutoff, Sequential merge cutoff	4096, 128, 4096
SparseLU	Block size	64
Strassen	Multiply by divide and conquer cutoff, Depth cutoff	128, ∞
UTS	Probability of non-leaf node, Number of children for non-leaf node, Root seed, Compute granularity	0.45, 2, 42, 50

**Fig 5:**
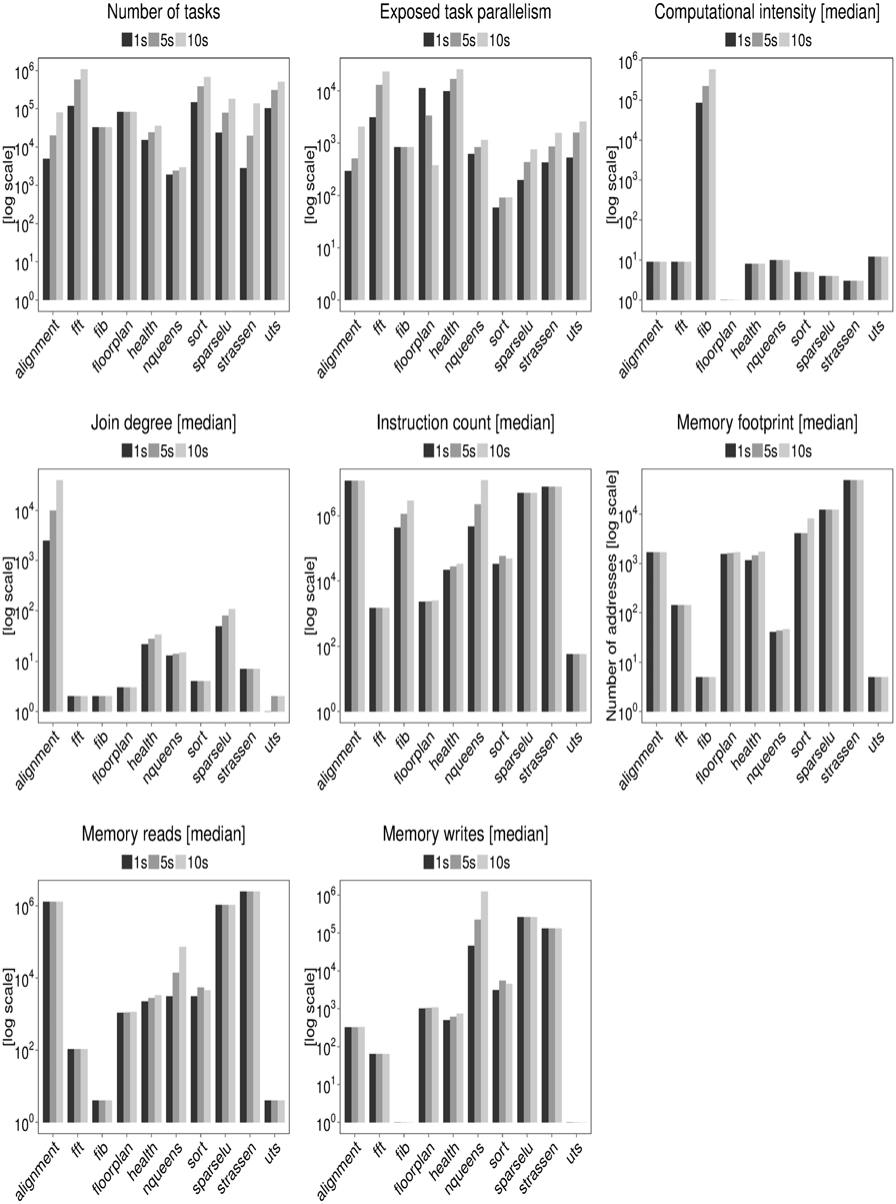
Task graph properties and per-task properties of BOTS benchmarks. 1s, 5s and 10s indicate execution time in seconds on 24-core AMD Opteron 6172 machine.

We show a subset of data collected by the input sensitivity experiment in Fig 5. We use median values to account for the most common change observable in extracted properties excluding outliers.

We validate input behavior as described in the previous section using the input sensitivity data in Fig 5. A summary of the validation is available in Table 6. Alignment, FFT, Sort, SparseLU, Strassen and UTS support the explanation by showing an increase in tasks and exposed parallelism with increasing data and constant GRAIN SIZE input. Fibonacci, Floorplan, Health and NQueens support the explanation by showing an increase in task grain size and holding exposed parallelism constant with increasing data and constant DEPTH. The number of tasks in Health and NQueens surprisingly increases with data size despite constant DEPTH input. The increase in tasks is explained by the increase in join node degree indicating a non-linear widening of the task graph.

**Table 6:** Summary of BOTS input sensitivity study. The symbol ↑ quantifies increasing, ↓ decreasing and → fixed input values.

**Benchmark**	**Input**	**Behavior**	**Scalability Estimate**	**Scalability Problems**
Alignment	Data ↑ GRAIN SIZE →	Tasks ↑ Exposed task parallelism ↑	Good	None
FFT	Data ↑ GRAIN SIZE →	Tasks ↑ Exposed task parallelism ↑	Worse	Poor memory hierarchy utilization, low computational intensity
Fibonacci	Data ↑ DEPTH →	Task grain size ↑ Exposed task parallelism →	Good	None
Floorplan	Data ↑ DEPTH →	Task grain size ↑ Exposed task parallelism ↓	Uncertain	Non-deterministic by construction
Health	Data ↑ DEPTH →	Task grain size ↑ Tasks ↑ Exposed task parallelism → Join degree ↑	Worse	Poor memory hierarchy utilization, low computational intensity
NQueens	Data ↑ DEPTH →	Task grain size ↑ Tasks ↑ Exposed task parallelism → Join degree ↑	Good	None
Sort	Data ↑ GRAIN SIZE →	Tasks ↑ Exposed task parallelism ↑	Worse	Poor memory hierarchy utilization, low computational intensity, low exposed task parallelism
SparseLU	Data ↑ GRAIN SIZE →	Tasks ↑ Exposed task parallelism ↑	Bad	Poor memory hierarchy utilization
Strassen	Data ↑ GRAIN SIZE →	Tasks ↑ Exposed task parallelism ↑	Bad	Poor memory hierarchy utilization
UTS	Data ↑ GRAIN SIZE →	Tasks ↑ Exposed task parallelism ↑	Worse	Poor memory hierarchy utilization, low computational intensity

We can make first-order estimates of the scalability of BOTS benchmarks using extracted task graph and per-task properties. A summary of the scalability estimates is available in Table 6. Fibonacci, Alignment and NQueens expose a large number of tasks with relatively high computational intensity and instruction count. The composition is favorable for performance since memory hierarchy utilization is likely to be high and parallelization overheads low. The benchmarks scale linearly on many architectures [8, 19] which validates our estimation. The benchmarks additionally expose a high and growing amount of exposed parallelism favoring scaling on larger machines.

FFT, Health, Sort and UTS create the largest number of tasks but with relatively low computational intensity and instruction count. The composition is ill-suited for performance since both memory hierarchy and the runtime system are likely to be stressed. Several studies have reported that the benchmarks scale poorly [8, 10, 20]. FFT, Health and UTS expose a high and growing amount of exposed parallelism which promises good scalability provided memory hierarchy utilization problems are solved. Sort is a pathological benchmark whose scaling will remain poor on larger machines due to a low amount of exposed parallelism. We show later that Sort’s low amount of parallelism is by construction.

SparseLU and Strassen expose high exposed parallelism using a large number of tasks with high instruction counts. However, low computational intensity of tasks is likely to stress the memory hierarchy. Poor scaling of the benchmarks when memory hierarchy effects are ignored have been reported [10, 18].

The decrease in exposed parallelism of the Floorplan benchmark with increasing data reflects its non-deterministic nature. Constant number of tasks yet a slow growth of instruction count are additional odd behaviors of the benchmark. The performance implications of Floorplan can only be understood by detailed experimentation.

### Similarity Analysis

We identify BOTS benchmarks with similar task graph properties and per-task properties using *Principal Component Analysis* (PCA) and *Hierarchical Clustering*—techniques commonly used to identify similarity of computer architecture workloads [21–23]. Our similarity analysis provides new directions to extend BOTS and pin-points redundant benchmark-input pairs which when avoided expedites timing-intensive studies such as computer architecture simulation.

Our experimental setup to measure similarity across BOTS benchmarks is inspired by the work of Eeckhout et al. [24] who meticulously describe their experimental setup to measure similarity between SPECint95 benchmarks.

We chose the following 10 task graph properties and per-task properties to measure similarity: number of tasks, memory footprint, memory reads, memory writes, stack reads, stack writes, instruction count, join degree, critical path and sequential work. The properties represent program execution in an independent manner—they cannot be directly derived from each other. Note that we avoided thread-based properties since they indistinguishably mix architecture and runtime system scheduler properties with program execution. Our intention is to measure similarity inherent in the task-based structure of BOTS benchmarks.

We profiled all 30 benchmark-input pairs in Table 4 and collected median values of the 10 properties into a 10×10×3 dataset called BOTS-INP-ALL. We *autoscaled* (scaling data to produce unit variance, zero-centered mean) BOTS-INP-ALL to assign equal weights to properties with different units. We performed PCA on BOTS-INP-ALL using the *princomp* function in the R statistical programming language and retained Principal Components (PCs) that accounted for 85% of variations in the data set. We analyzed the meaning of the retained PCs and projection of BOTS-INP-ALL in the retained PC space.

We autoscaled retained PC scores and applied agglomerate hierarchical clustering using the *hclust* function in R with Euclidean distance as the linkage metric and Ward’s minimum variance method for clustering data. We visualized the result of hierarchical clustering in a dendogram and cut groups into it to infer similarity.

**Fig 6:**
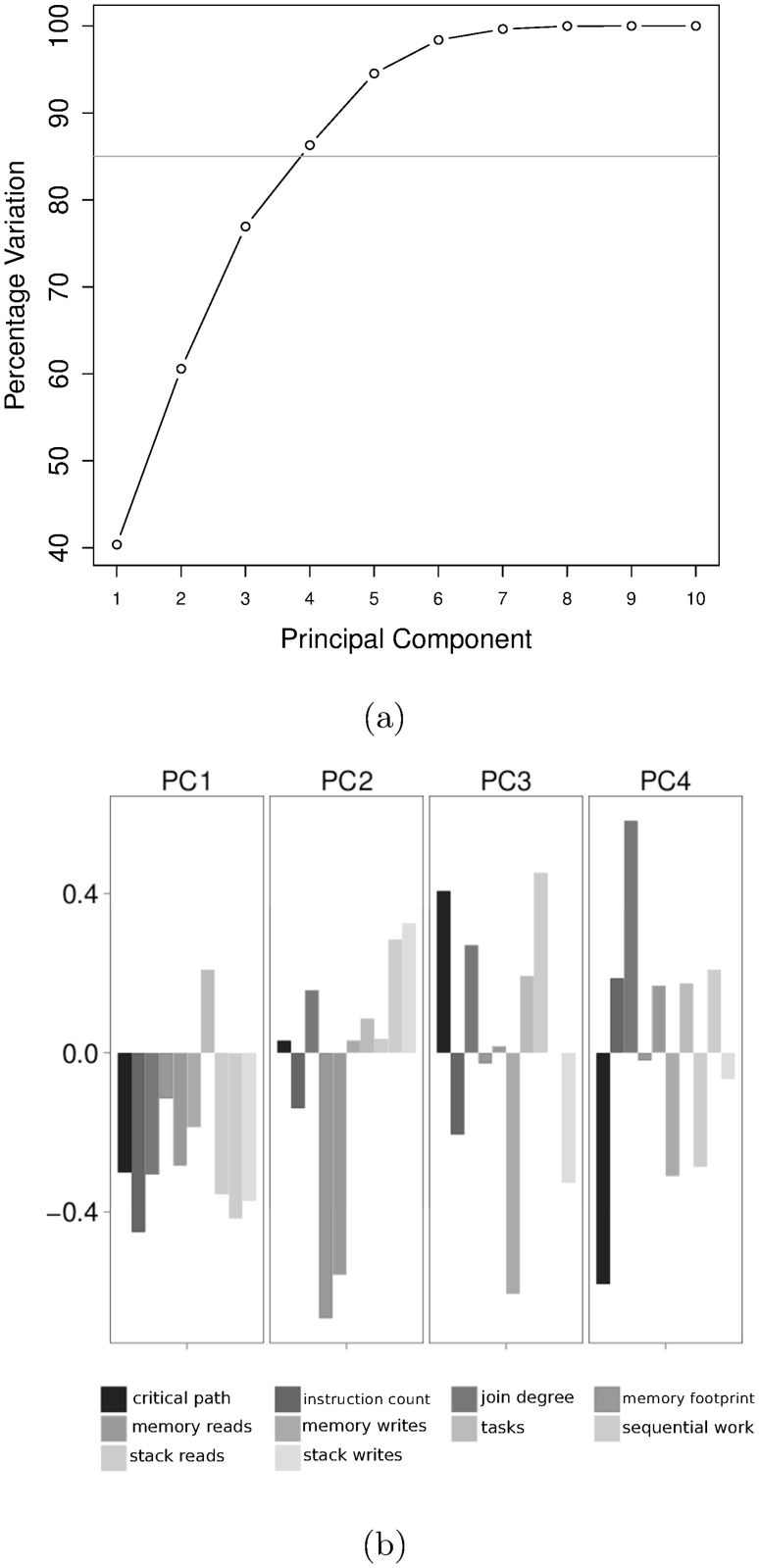
Principal Component Analysis. (a) Variation explained by Principal Components. We retain the first four Principal Components which explain more than 85% variation. (b) Loadings of retained Principal Components.

We can account for more than 85% of variations in BOTS-INP-ALL using the first four PCs as shown in Fig 6a.

Component loadings of the four retained PCs are shown in Fig 6b. PC1 is an indicator of fine-grained benchmarks since it is positively influenced by the number of tasks and negatively influenced by instruction count. PC2 indicates benchmarks with stack memory intensive tasks, PC3 those with limited parallelism and fine-grained tasks and PC4 those benchmarks with limited parallelism, large-grained tasks implemented using iterative fork-join parallelism.

**Fig 7:**
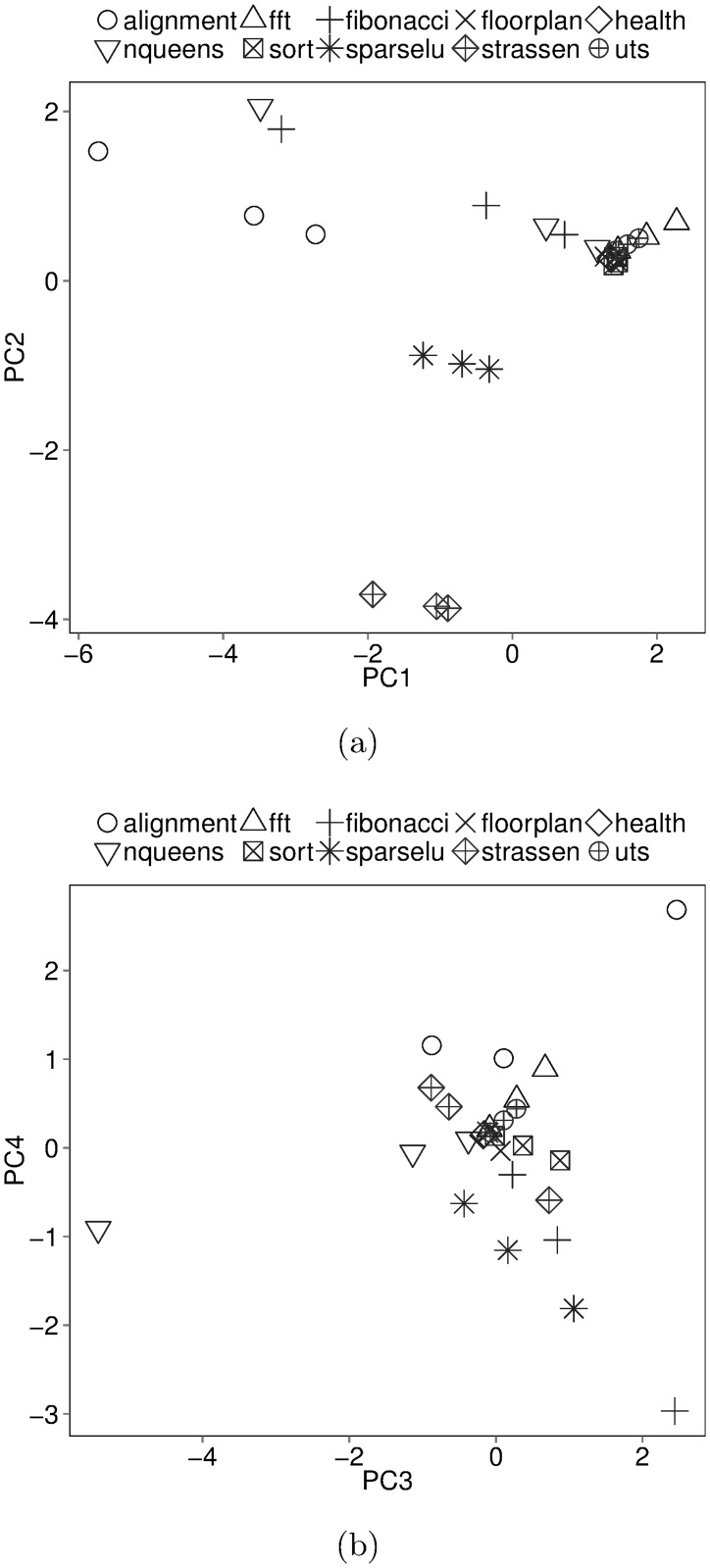
Projection in retained Principal Component space.

Planes PC1-PC2 and PC3-PC4 defined by projecting benchmark-input pairs into the four-dimensional retained PC space are shown in Fig 7. Alignment, NQueens and Fibonacci show widely dissimilar behavior with increasing data sizes since they are spread out in both planes. Strassen and SparseLU show slightly dissimilar behavior. The rest—Health, Sort, UTS, Floorplan and FFT form close clusters indicating similar behavior irrespective of data size. Experiments using Health, Sort, UTS, Floorplan and FFT can save evaluation time by using the smallest data size. The empty spaces in the PC1-PC2 plane indicate an abundance of fine-grained but few memory intensive benchmarks in BOTS.

**Fig 8:**
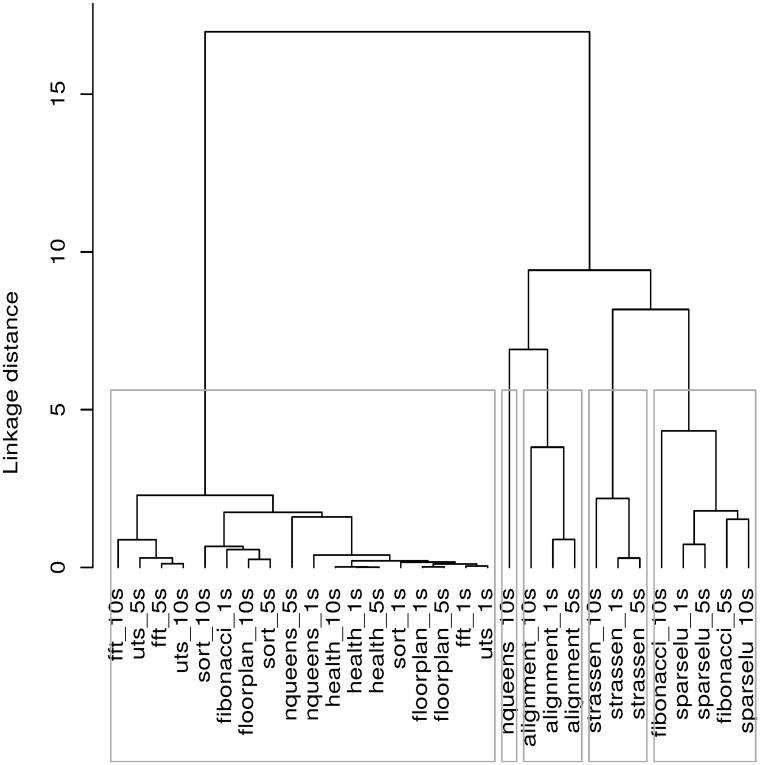
Hierarchical clustering dendogram with a 5-cluster cut.

Results of hierarchical clustering of BOTS-INP-ALL are summarized in Fig 8. The large cluster on the left shows that several benchmarks behave similarly not only across data inputs but also across each other. The remaining clusters indicate benchmarks with similar properties across data inputs. We can conclude that benchmark-input pairs in BOTS-INP-ALL have a strong resemblance in terms of the task-based properties chosen in our experiment. The similarity allows researchers concerned mainly with our chosen set of task-based properties to pick a single benchmark from each cluster and save evaluation time in timing-intensive experiments.

We reduced the likelihood of over-fitting by taking several measures. We have ensured that the BOTS-INP-ALL is complete without any missing values. We have reduced the number of variables describing data in two steps. First, we chose 10 independent task graph properties and per-task properties while composing the BOTS-INP-ALL dataset. Next, we scored BOTS-INP-ALL using 4 PCs that described more than 85% of variations. Furthermore, we avoided bias by autoscaling both initial and scored datasets. As a last step to avoid over-fitting, we performed *leave-one-out* cross-validation on BOT-INP-ALL which estimated that 4 PCs can describe the changing datasets with minimal error. We performed leave-one-out cross-validation using the R function *estim_ncpPCA* part of the *missMDA* package.

## Diagnosing Performance Problems

We demonstrate how task-based performance can be used to diagnose performance problems quickly and understand performance tradeoffs using two troublesome programs—BOTS Sort and Strassen—as examples.

### Sort

The benchmark sorts a given array of numbers by breaking it recursively into progressively smaller chunks and applying different sorting algorithms on individual chunks. Sorted chunks are recursively merged. The choice of sorting algorithm and merge recursion depth is guided by multiple independent chunk sizes provided as inputs called *cutoffs*.

**Fig 9:**
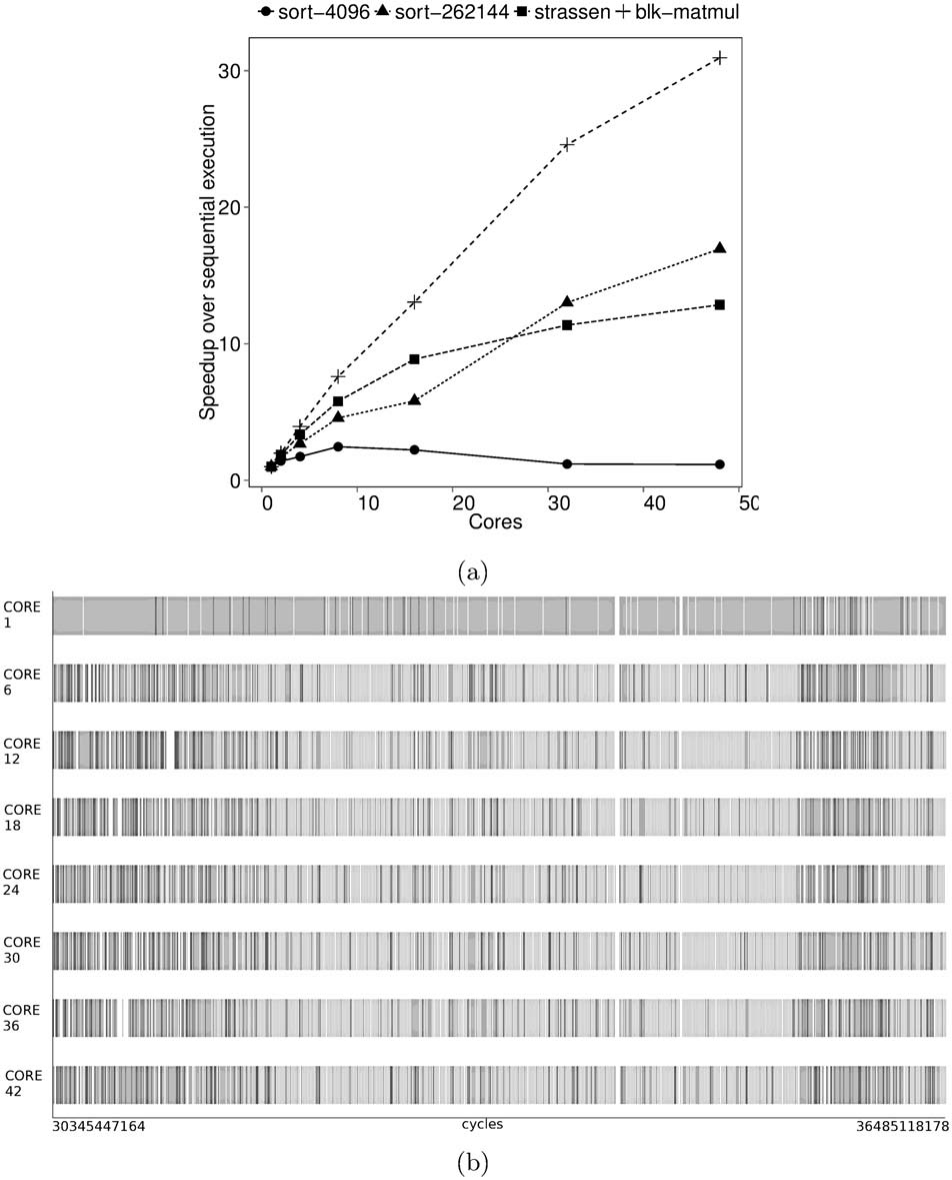
Diagnosing performance problems using thread-based performance metrics in BOTS Sort and Strassen. Sort input: array size = 64M elements, quicksort cutoff = {4096 (default), 262144}, sequential merge sort cutoff same as quicksort cutoff, insertion sort cutoff = 128. Strassen input: dimension = 4096, cutoff = 128 (default). Blocked matrix multiplication (blk-matmul) input: dimension = 4096, block size = 128. Executed on all cores of 48-core AMD Opteron 6172 machine running at highest frequency with frequency scaling turned off. (a) Speedup (b) Visualization of state traces from 6/48 threads executing Sort with default cutoffs. White bars indicate task creation, black bars, task execution and gray bars, task synchronization. The six threads are bound to cores on different dies.

Sort performs badly under default cutoffs as shown in Fig 9a. However, performance improves significantly when good cutoffs are provided.

Thread-based performance cannot explain performance adequately. Thread state durations show more time spent in parallelization than in execution of tasks under default cutoffs, hinting without quantification that tasks are fine-grained. We cannot ensure if overall parallelization time is a robust measure i.e., if parallelization time surpasses execution time of most tasks or just a few. Inspecting thread time-lines shown in Fig 9b shows that tasks are created more frequently than executed but cannot explain why. Moreover, the inspection process requires careful navigation through dense time-lines—a difficult process when dealing with a large number of threads. Manual-tuning for multiple independent cutoffs is the only option available given thread-based performance.

**Fig 10:**
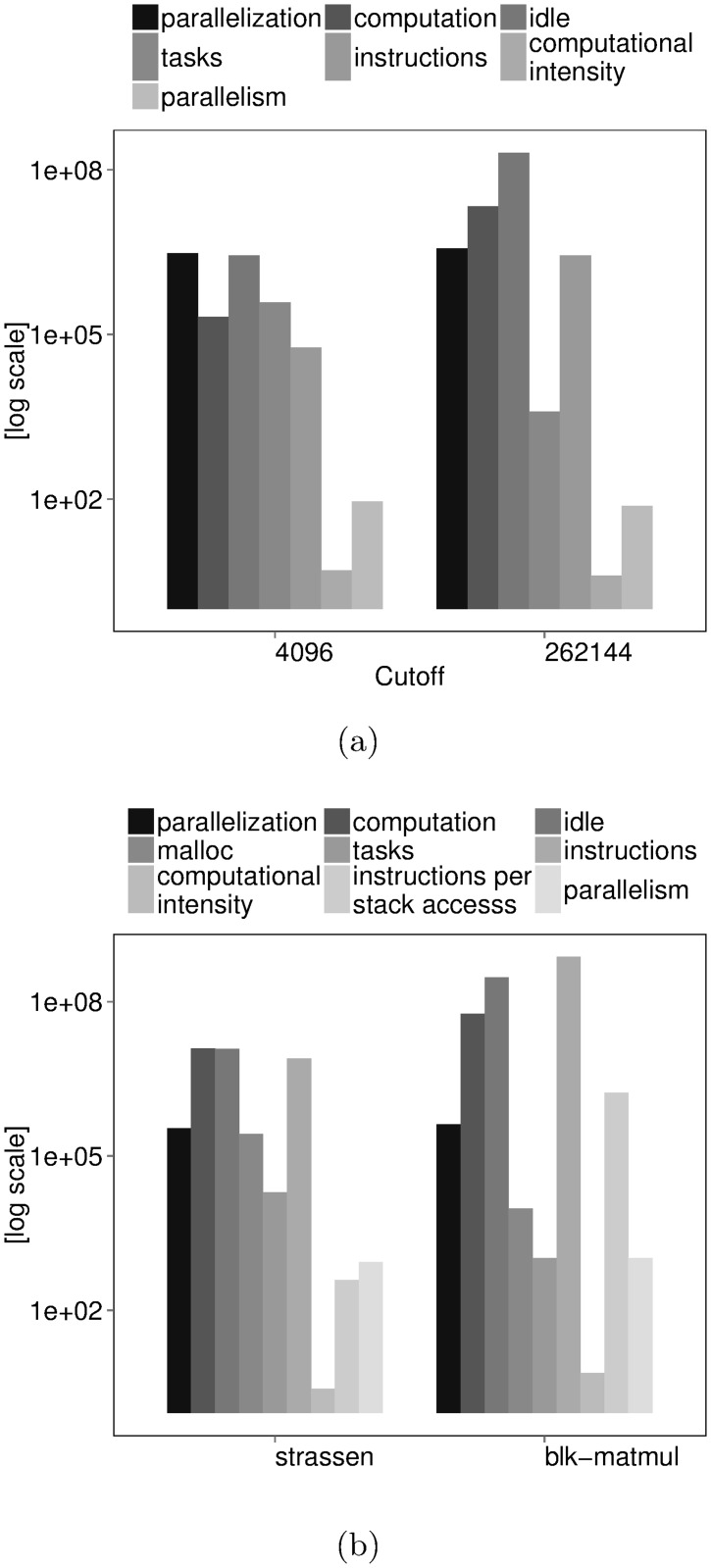
Diagnosing performance problems using task-based performance metrics in BOTS Sort and Strassen. Sort input: array size = 64M elements, quicksort cutoff = {4096 (default), 262144}, sequential merge cutoff same as quicksort cutoff, insertion sort cutoff = 128. Strassen input: dimension = 4096, cutoff = 128 (default). Blocked matrix multiplication (blk-matmul) input: dimension = 4096, block size = 128. Executed on all cores of 48-core AMD Opteron 6172 machine running at highest frequency with frequency scaling turned off. Time spent in parallelization, computation and idle states is expressed in cycles. (a) Sort cutoff performance. (b) Strassen and Blocked matrix multiplication (blk-matmul) performance.

Task-based performance shown in Fig 10a can diagnose performance conclusively. We can confirm that per-task execution time is undoubtedly lower than parallelization time under default cutoffs. Large per-task idle time indicates load imbalance on threads. Exposed parallelism cannot compensate exceeding parallelization costs despite being higher than the available hardware parallelism. Low computational intensity indicates an algorithmic problem in the computation assigned to tasks.

**Fig 11:**
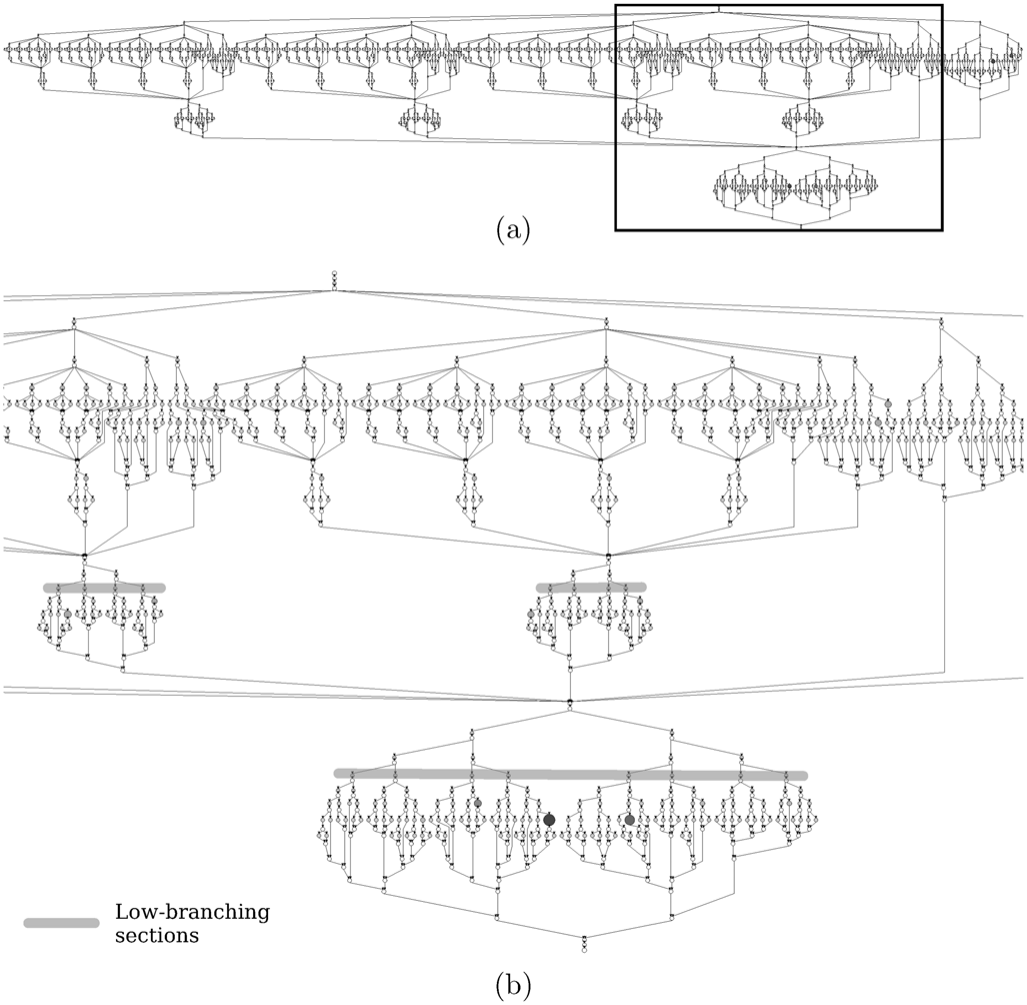
Task graph of BOTS Sort. Input: array size = 4096 elements, quicksort cutoff = sequential merge cutoff = 32. Input sizes are reduced to obtain graphs which satisfy space restrictions and preserve clarity. (a) Task graph with rectangle marking area of interest. (b) Task graph zoomed into area of interest.

Inspecting the task-graph produced by our tool further reveals performance bottlenecks. Exposed parallelism is non-uniform under default cutoffs as shown in Fig 11. Parallelism grows gradually from the top and becomes highest in the middle of the task-graph as is typical for recursive fork-join task-based programs. However, unlike the typical case, Sort continues to work exposing non-uniform parallelism while winding down which creates the load imbalance seen in thread-based measurements. The task graph also points to aggressive branching as the reason behind frequent task creation phases seen on thread time-lines.

Task-based performance can also improve program understanding and expedite tuning. Let us try to reduce the high task creation frequency under default cutoff conditions. Code-links associated with fork edges of our task graph reveal that the cutoffs themselves are responsible for task creation. The implication of cutoffs becomes clear when we link fork points to responsible cutoffs using the captured data environment. We can immediately get good performance by selecting cutoffs in the low-branching sections of the task graph. Note that the same cutoffs emerge from manual-tuning.

Task-based performance under good cutoffs is shown in Fig 10a. Task execution time exceeds parallelization time and is higher than the default cutoffs counterpart. Idle time remains high due to non-uniform parallelism and large-grained tasks. Parallelism reduces slightly compared to the default cutoffs counterpart but is large enough to match the available hardware parallelism. Low computational intensity points to a continued algorithmic problem in task computation.

### Strassen

The benchmark multiplies matrices by recursively splitting them into progressively smaller blocks. The smallest blocks are multiplied using the Strassen multiplication method. Tasks are used to split blocks until they reach a cutoff size provided as input. Blocks are split using sequential execution after the task cutoff point.

**Fig 12:**
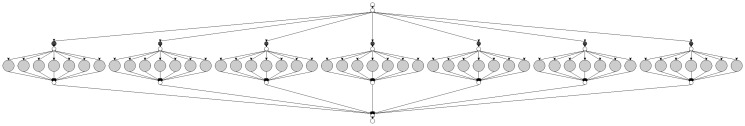
Task graph of BOTS Strassen. Input: dimension = 512, divide and conquer multiplication cutoff = 128, depth cutoff = ∞. Input sizes are reduced to obtain graphs which satisfy space restrictions and preserve clarity.

Strassen scales modestly in our test case shown in Fig 9a. Task-based metrics can help boost performance further. Per-task execution time in Fig 10b exceeds parallelization time suggesting that performance problems lie elsewhere. Low computational intensity, frequent stack accesses and a large amount of time spent in dynamic memory allocation during task execution point to an algorithmic problem. The task graph in Fig 12 reveals work concentration at leaf tasks confirming the algorithmic problem.

We solved the algorithmic problem by re-implementing the leaf task computation as a BLAS-enabled blocked matrix multiplication. Our strategy improves performance significantly as shown in Fig 9a at the one-time cost of finding a cache-friendly block size.

## Related Work

### Performance Analysis Tools

Popular OpenMP profiling tools Intel VTune Amplifier [2, 3], Sun Studio Performance Analyzer [6], Scalasca [7], Vampir [25] and HPCToolKit [5] capture detailed thread state and event performance but do not attribute captured performance to tasks [4].

Few profiling tools support capturing and presenting OpenMP task performance to the user. A common advantage of our tool is richer task performance information obtained at lower costs.

Qian et al. [26] combine the thread line and the function call graph to construct a hybrid graph called the Profiled Timeline Graph (PTG) which shows time spent by threads executing tasks and scheduling points of OpenMP programs. While the PTG is useful to infer runtime system overheads and task granularity, it cannot measure exposed task parallelism—a crucial metric to diagnose performance problems. Our method characterizes exposed task parallelism, per-task hardware and instruction-level performance in addition to metrics indicated by the PTG at lower profiling costs. Our implementation lowers profiling costs by tightly coupling with the runtime system and using parallel post-processing whenever possible. The PTG implementation suffers high overheads since it uses source instrumentation, is decoupled from the runtime system and uses sequential post-processing.

Ding et al. [27] retrieve parent-child relationship and execution time of tasks from OpenMP programs. The authors use the information to construct a visual fork-join task graph similar to our approach. Their fork-join task graph connects fork and join edges from children to the same parent node, forming cycles which makes calculation of exposed task parallelism difficult and complicates graph layouting. Our fork-join task graph separates fork and join nodes from task nodes and joins them to form a DAG which simplifies both parallelism calculation and graph layouting. The authors also suggest super-imposing fork-join information with thread time-line visualization which reduces clarity for large thread counts. Their tool is implemented as an external library that perform task monitoring by intercepts calls to the libgomp runtime system. Compared to our method which is implemented in the runtime system itself, an external library allows decoupling from the runtime system implementation which potentially reduces implementation complexity. However, the external library approach is restricted to capture information at interception points or events which makes it hard to capture non-events such as task completion and task switching. Task completion and task switching events are necessary to attribute per-task properties to each task instance. Another drawback of external monitoring is fragility to runtime system changes. Their tool profiles parent-child relationships and task execution time at an average 15% overhead for BOTS benchmarks whereas our implementation extracts the same metrics incurring an average 2.5% overhead.

The OmpP tool [28] uses source instrumentation to record entry into, execution time within and exit from OpenMP task regions but without not per-task information which is necessary to study performance of tasks in isolation. Our methods provide richer performance information than OmpP.

Lin and Mazurov [29] extend OpenMP performance sampling API [30] with support for querying the runtime system for task execution information such as task instance identification and parent-child relationships. Use of such information in performance analysis is not demonstrated in their paper. Qawasmeh et al. [31] implement OpenMP performance sampling API for task-based profiling. They capture creation, execution and synchronization time of individual task instances with low-overheads similar to our approach, but do not demonstrate how captured information can be used to solve performance problems. Our methods additionally capture exposed task parallelism and per-task hardware and instruction-level performance, and demonstrate their usefulness in improving real-world OpenMP programs.

OMPT [32, 33] is a performance analysis API recently proposed by the OpenMP Tools Working Group. OMPT allows external tools to asynchronously sample execution states of and register for event notification with the runtime system. Our approach to uniquely identify task instances and track their creation, execution and synchronization can also be performed using task-centric data structures and notification mechanisms specified by OMPT. While OMPT covers all aspects all aspects of OpenMP execution such as parallel regions, work-sharing for-loops and tasks, our approach focuses on task-based execution. OMPT is well thought-out but is presented surprisingly without an accompanying implementation, complicating overhead comparison. Demonstration of how OMPT supplied information can be used to solve performance problems in OpenMP programs is also missing in OMPT literature. OMPT is incapable of characterizing exposed task parallelism and per-task hardware and instruction-based performance.

The debugger IDB [34] in Intel Composer XE 2013 provides task creation and synchronization information but not task execution time for OpenMP programs. Task execution time is crucial for performance tuning in which tasks are composed to be larger than runtime system overheads but not too large to cause load imbalance. Intel also provides an autotuner called the Intel Software Autotuning Tool [35] which can graphically quantify relationships between task granularities and inputs of TBB programs.

The Score-P profiling system [36] uses source instrumentation [37] to track and attribute execution time to OpenMP task instances. Score-P visually depicts a call-graph structure that indicates time spent executing tasks and scheduling points. The authors demonstrate utility of Score-P by using task creation overhead measurements to pick good cutoff values for the BOTS NQueens benchmark. We similarly demonstrate usefulness of task-based performance analysis by picking good cutoff values for the BOTS Sort benchmark. Schmidl et al. [38] embed profiling information from Score-P into thread time-line visualization to simplify detection of improper task granularities and high task creation overheads. In our experience, viewing Score-P metrics using the CUBE tool from Scalasca [7] to count the number of tasks created and executed by threads is the most advanced and user-friendly task-based analysis support available in state-of-the-art tools. However, support for exposed task parallelism calculation and profiling per-task hardware and instruction-level performance is absent in Score-P and its derivatives [4].

The Cilkview tool [9] monitors *logical parallelism* and analyses the scalability of Cilk Plus programs. Logical parallelism is an intrinsic, construction-based property of Cilk Plus programs which we adopt to derive the notion of exposed task parallelism in task-based OpenMP programs. Cilkview extracts critical path of the task graph using instruction count as a proxy for execution time similar to our approach.

Aftermath [39] is a graphical tool to analyze performance problems in programs written using OpenStream, a streaming/data-flow task-based programming language. Aftermath applies a combination of task-based and topology-based filters on thread time-line visualization to aggregate task execution times, per-task memory performance and per-task communication patterns. It is unclear whether Aftermath supports filtering and aggregation of recursive tasks which are the most common type of tasks composed in OpenMP programs. Aftermath cannot quantify exposed task parallelism and per-task instruction-level performance.

### BOTS Characterization

While thread-based performance of BOTS benchmarks has been extensively characterized on several multicore architectures and under different scheduler implementations [8, 10, 19, 40–42], only few task-based characterizations of BOTS benchmarks exist.

BOTS creators guide runtime system design using average values of per-task instruction count and memory accesses profiled for the medium input set [8]. Lorenz et al. [36] use Score-P to profile and report mean task execution time for BOTS benchmarks for the medium input set. Ding et al. [27] provide task creation and synchronization counts of BOTS benchmarks for a custom input set. Qian et al. [26] provide task count, average task execution time and creation time (GCC 4.7.2) of BOTS benchmarks for a custom input set. Input sensitivity studies of BOTS benchmarks using task-based performance measurements are absent. Similarity analysis of BOTS benchmarks using both thread-based and task-based performance measurements are also absent.

We focus on techniques that guide proper choice of *cutoff* inputs to increase performance of task-based programs in the paper. Cutoffs inhibit task creation for performance and are currently inferred using manual tuning and expert judgment. Automatic choice of good cutoffs in the runtime system has been studied by Duran et al. [43]. They use dynamically profiled execution time of past tasks to adaptively inhibit or allow task creation and obtain good performance for task-based OpenMP programs that predate BOTS. Although wise cutoff support in the runtime system is desirable, widely-used compilers GCC (version 4.9) and Intel ICC (libomp_oss version 20131209) implement simple heuristic-based cutoffs. GCC cuts off task creation when the number of tasks in the system exceeds a magic number (number of threads*64). ICC cuts off task creation when tasks queues are full. Both mechanisms did not work well with BOTS benchmarks in our tests allowing us to conclude that proper cutoff choice continues to be a programmer responsibility.

## Conclusions

We have provided a simple, automated method to extract detailed task-based performance from OpenMP programs at manageable costs. Using our method, we contribute with an extensive, architecture independent characterization of task parallelism in BOTS using a prototype implementation of our method. Our characterization complements existing thread-based BOTS characterizations with a task-based explanation of inputs and architecture independent performance implications. We have shown that several BOTS benchmarks exhibit similar architecture independent behavior which guides researchers when selecting BOTS inputs and saves evaluation time. We have demonstrated how task-based performance can be used to diagnose performance problems quickly and understand performance tradeoffs while analyzing OpenMP programs.
